# Systems Biology for Organotypic Cell Cultures

**DOI:** 10.14573/altex.1608221

**Published:** 2016-11-14

**Authors:** Sonia Grego, Edward R. Dougherty, Francis J. Alexander, Scott S. Auerbach, Brian R. Berridge, Michael L. Bittner, Warren Casey, Philip C. Cooley, Ajit Dash, Stephen S. Ferguson, Timothy R. Fennell, Brian T. Hawkins, Anthony J. Hickey, Andre Kleensang, Michael N. Liebman, Florian Martin, Elizabeth A. Maull, Jason Paragas, Guilin (Gary) Qiao, Sreenivasa Ramaiahgari, Susan J. Sumner, Miyoung Yoon

**Affiliations:** 1 RTI International, Research Triangle Park, NC, USA; 2 Texas A&M University, College Station, TX, USA; 3 Los Alamos National Laboratory, Los Alamos, NM, USA; 4 National Institute of Environmental Health Sciences, Research Triangle Park, NC, USA; 5 GlaxoSmithKline, Research Triangle Park, NC, USA; 6 Translational Genomics Research Institute, Phoenix, AZ, USA; 7 HemoShear Therapeutics, Charlottesville, VA, USA; 8 Johns Hopkins University, Center for Alternatives to Animal Testing, Baltimore, MD, USA; 9 IPQ Analytics, Kennett Square, PA, USA; 10 Phillip Morris International R&D, Neuchâtel, Switzerland; 11 Lawrence Livermore National Laboratory, Livermore, CA, USA; 12 Defense Threat Reduction Agency, Ft. Belvoir, VA, USA; 13 The Hamner Institutes for Health Sciences, Research Triangle Park, NC, USA; 14 ScitoVation, Research Triangle Park, NC, USA

**Keywords:** systems toxicology, systems pharmacology, multiscale modeling, 3D models, engineered cell cultures

## Abstract

Translating *in vitro* biological data into actionable information related to human health holds the potential to improve disease treatment and risk assessment of chemical exposures. While genomics has identified regulatory pathways at the cellular level, translation to the organism level requires a multiscale approach accounting for intra-cellular regulation, inter-cellular interaction, and tissue/organ-level effects. Tissue-level effects can now be probed *in vitro* thanks to recently developed systems of three-dimensional (3D), multicellular, “organotypic” cell cultures, which mimic functional responses of living tissue. However, there remains a knowledge gap regarding interactions across different biological scales, complicating accurate prediction of health outcomes from molecular/genomic data and tissue responses. Systems biology aims at mathematical modeling of complex, non-linear biological systems. We propose to apply a systems biology approach to achieve a computational representation of tissue-level physiological responses by integrating empirical data derived from organotypic culture systems with computational models of intracellular pathways to better predict human responses. Successful implementation of this integrated approach will provide a powerful tool for faster, more accurate and cost-effective screening of potential toxicants and therapeutics.

On September 11, 2015, an interdisciplinary group of scientists, engineers, and clinicians gathered for a workshop in Research Triangle Park, North Carolina, to discuss this ambitious goal. Participants represented laboratory-based and computational modeling approaches to pharmacology and toxicology, as well as the pharmaceutical industry, government, non-profits, and academia. Discussions focused on identifying critical system perturbations to model, the computational tools required, and the experimental approaches best suited to generating key data.

## 1 Introduction

### 1.1 Organotypic cell cultures

**Fig. 1:**
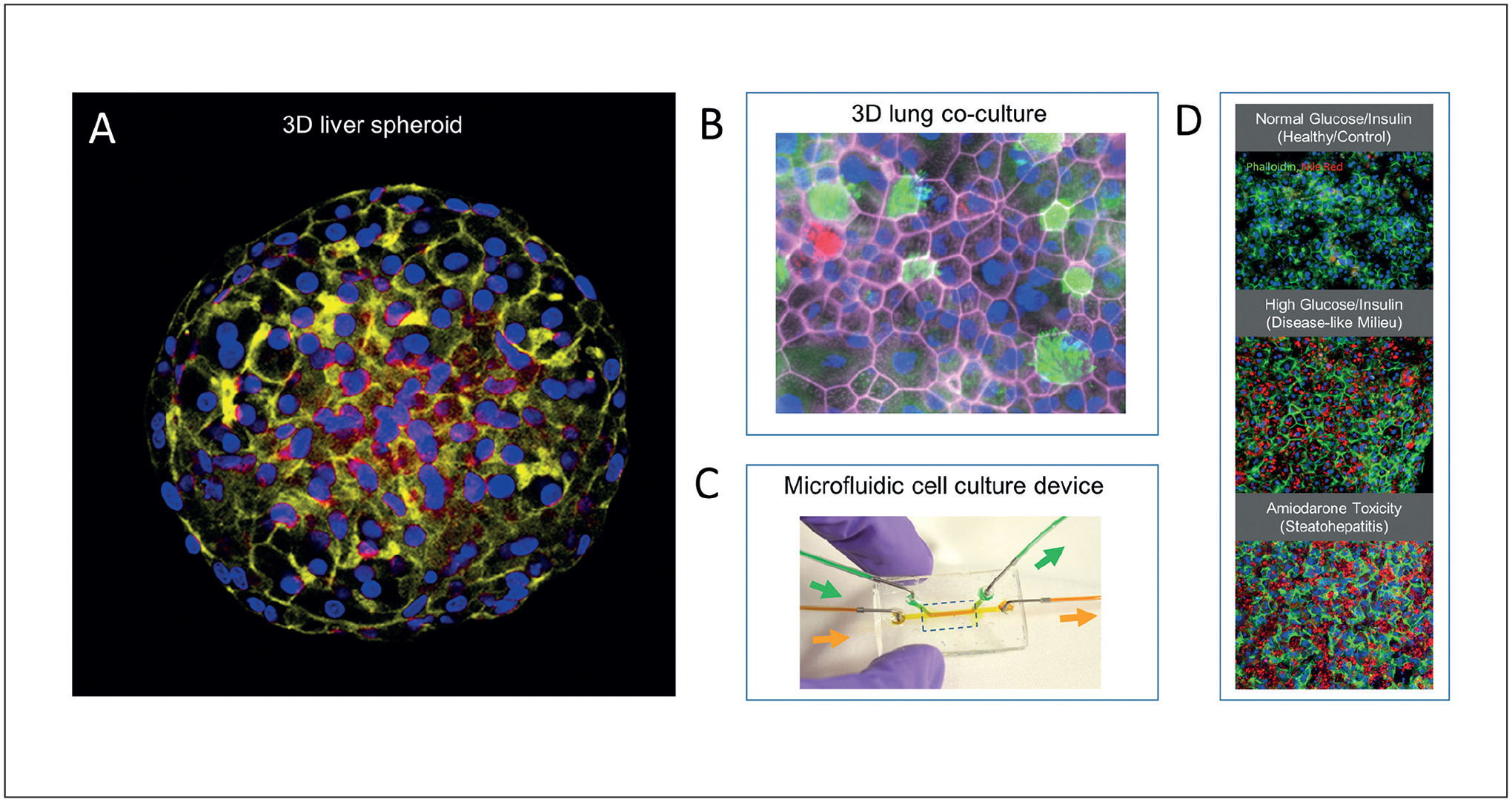
Examples of organotypic cell culture systems (A) HepaRG liver cell spheroid stained for actin cytoskeleton and nuclei (red = actin, blue = nuclei). (B) Primary airway epithelial cells grown at air-liquid interface (pink = actin, red = mucin, green = cilia, blue = nuclei) in a 3D co-culture with microvascular cells (not shown). (C) Micromolded microfluidic device for cell culture on a nanoporous membrane. (D) The HemoShear technology uses cone and flow viscometer principles to apply hepatic microcirculation flow and transport parameters over 2 weeks. Hepatocytes in this system can be cultured at physiological insulin concentrations (1000fold lower than in static cultures) resulting in retention of insulin sensitivity and responses. Hepatocytes develop steatotic changes (Nile Red staining for lipid) under high glucose/high insulin milieus, allowing modeling of disease-like states, and exhibit drug-induced toxicity phenotypes like steatohepatitis when treated with therapeutic concentrations of amiodarone. (Panel A courtesy of S. Ferguson, NIEHS/NIH; Panels B-C courtesy of S. Grego, RTI International; Panel D courtesy of A. Dash, HemoShear)

An intense area of research aims at improving drug development and toxicology assessment by shifting away from traditional animal studies and towards human relevant, *in vitro* systems for accurate and cost-effective prediction of human health outcomes. Development of increasingly sophisticated *in vitro* tissue models has accelerated in recent years, driven by the recognition that conventional two-dimensional (2D) cell culture formats do not adequately recapitulate the 3D arrangements of cells and extracellular matrix of tissues and organs (Andersen et al., 2014; Dalton and Dougherty, 2013, 2014). The loss of these mechanical and spatial cues in culture alters the physiology and function of cells (Huh et al., 2012a). To address these limitations, more physiologically complex and relevant cell culture formats have been produced through biomedical engineering applications and are variously referred to as “organotypic”, “organoid”, or, when microfabrication techniques are used, “organ-on-a-chip” or “microfluidic” cell culture systems (Benam et al., 2015; Huh et al., 2012b). These engineered cell culture systems supported by advances in human-derived stem cells, aim at developing new tools for broad applications in preclinical drug testing and toxicant screening, and have been supported by substantial funding initiatives. Agencies in the U.S., including DARPA (Defense Advanced Research Projects Agency), DTRA (Defense Threat Reduction Agency), NIH (National Institutes of Health) and EPA (U. S. Environmental Protection Agency) committed almost $200 million starting in 2011 (Wikswo, 2014). The European Union FP7 funded the “Safety Evaluation Ultimately Replacing Animal Testing” (SEURAT) program (Daston et al., 2015), which with funding matched by the cosmetic industry, achieved €50 million in 2011–2016. This was followed by the €30 million EU-ToxRisk collaborative project (2014–2020) (Daneshian et al., 2016). Many successful examples of organ/tissue models have been recently reported (Fig. 1) (Adler et al., 2015; Alépée et al., 2014; Andersen et al., 2014; Dash et al., 2009, 2013; Hawkins et al., 2015; Hogberg et al., 2013; Mortensen et al., 2016; Pamies et al., 2014; Sarkar et al., 2015; Sellgren et al., 2014, 2015; Smirnova et al., 2015; Terelius et al., 2015; Torisawa et al., 2014; Walter et al., 2016; Wikswo, 2014).

### 1.2 The need for computational analysis of data from organotypic model systems

Despite the great strides made in terms of engineered micro-environments and novel human cell sources supporting development of more physiologically relevant culture models, the integration, analysis, modeling, and ultimately application of the data generated in such complex model systems remains a significant challenge for the research community.

The field of organotypic cultures seeks ultimately to support more patient-relevant drug safety and efficacy testing, and human-relevant toxicity screening, mainly by using induced pluripotent stem (iPS) cell techniques to produce patient- and population-specific organs-on-chips (Esch et al., 2015; van Duinen et al., 2015). While a multiplicity of organotypic cultures has been shown to better recapitulate tissue features (Mortensen et al., 2016), there remain the key hurdles of validating these constructs for safety and efficacy screening and of their widespread adoption to obtain human-relevant and actionable data. This requires a computational approach that reliably extrapolates from tissue models to whole organism responses (IVIVE: *in vitro* to *in vivo* extrapolation) and provides testable predictions.

### 1.3 Systems biology and computational modeling

Computational systems biology approaches aim to accurately and predictively model biological systems by applying mathematical tools developed for complex, nonlinear systems:

- Nonlinearity is inherent in biology because cell regulation and responses to perturbation – while both concentration- and time-dependent – involve multiple biochemical pathways that may have common nodes and involve parallel, redundant, and feedback loop processes.
- Complexity derives from several factors: the number of genes and regulatory pathways, multivariate regulation, and the range of adaptive physiology and responses over a variety of scales, both spatial (from molecules to cells to tissue to organ) and temporal (from seconds for molecular signals to years for degenerative diseases).

Cell health and function are under the control of complex, dynamic regulatory processes at the gene, protein and pathway levels. To ensure survival under dynamic micro-environmental conditions, normal cellular physiology is highly adaptable. Perturbations to these systems (disease, drugs, or toxicants) can overcome normal adaptive mechanisms resulting in maladaption and pathology/toxicity (Kleensang et al., 2014). The progression from homeostatic cellular physiology to adaptive physiology to pathophysiology can be characterized quantitatively by measures of gene or protein expression, cellular function, or morphologic phenotype.

With the advent of high-throughput technologies and high-content assays, a systems-oriented approach to biological sciences is emerging that represents a shift from the classical reductionist approach. Systems biology approaches have been developed to analyze large data sets provided by genomics, transcriptomics, proteomics, metabolomics, and protein-protein interaction (PPI) networks (Auerbach et al., 2015; Bouhifd et al., 2013; Church et al., 2014; Ivanov et al., 2007; Kumar et al., 2015; Mohsenizadeh et al., 2015; Spirin and Mirny, 2003; Wang et al., 2014). The challenge is in the integration and interpretation of these complex datasets. In order to achieve mechanistic insight into the underlying biology, this may require *a priori* knowledge such as networks (e.g., causal models). Systems-wide data and cell type or tissue specific causal network models describing key biological mechanisms, such as cell stress, proliferation, inflammation, or cell fate can be integrated, enabling a systems biology-based quantitative interpretation of the underlying *in vitro* experiment (Martin et al., 2012, 2014; Thomson et al., 2013).

**Fig. 2:**
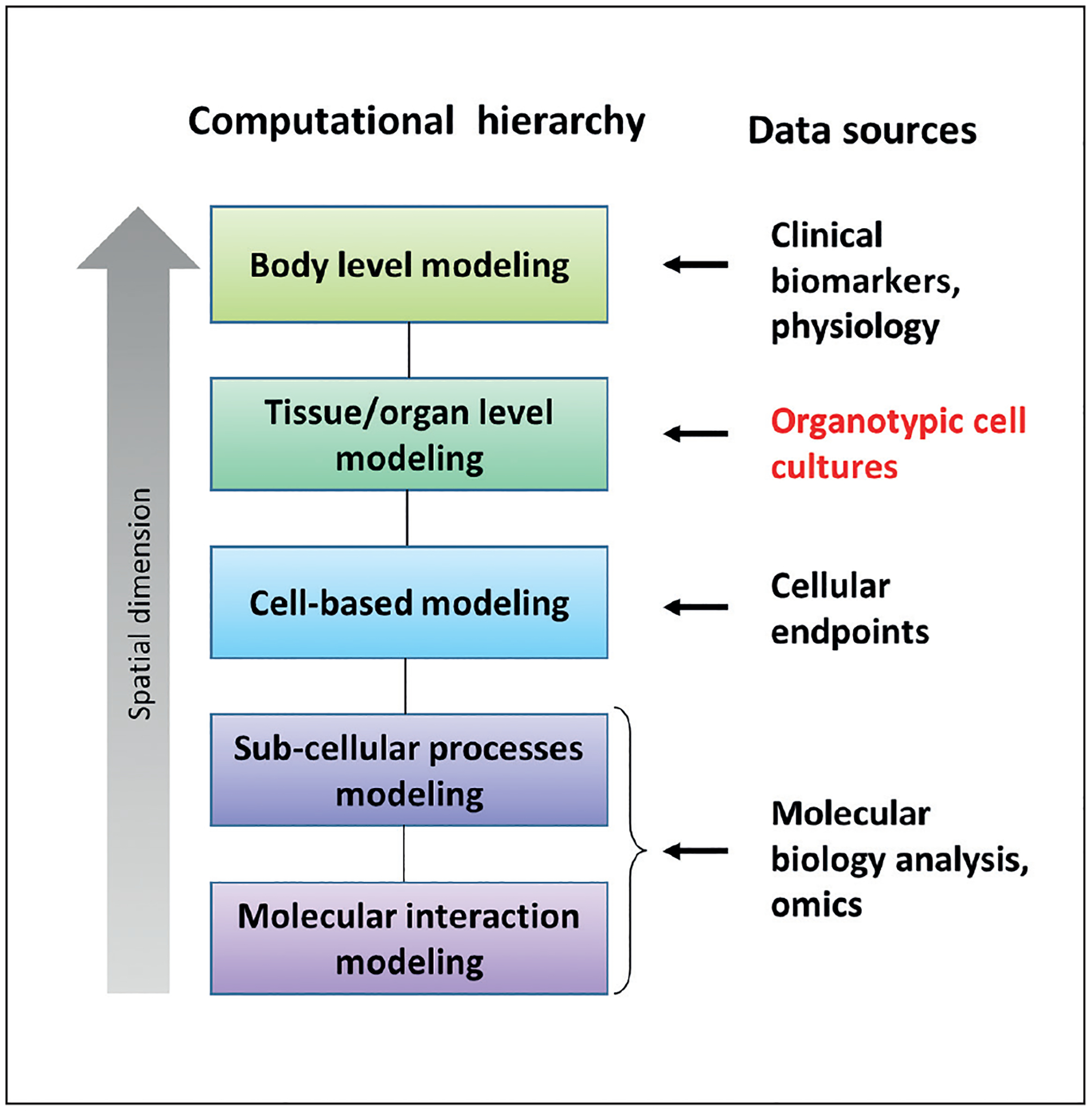
Outline of hierarchical structure of multiscale biological modeling Computational models exist for each of the level, having an integrated model linking the “spatial dimensions” is the real challenge.

For computational tractability, the number of model components derived from existing pathway knowledge and data must be kept small while still representing cellular activity with sufficient accuracy to be predictive. Network modeling approaches satisfying these constraints have been developed for individual cells; however, models representing the multiscale pharmacokinetic/pharmacodynamic processes required to describe biological responses at the tissue/organ/organism level (Fig. 2) are needed.

In the systems toxicology field, the recently introduced framework of Adverse Outcome Pathways (AOP) stipulates a holistic, systematic extrapolation from empirical data across spatial scales (Ankley et al., 2010). The AOP framework enables a systems-level understanding of exposure to a potentially toxic substance by describing a cascade of multiscale events from exposure to adverse effect, with the goal of a more effective risk assessment. A multiscale computational modeling approach to describing biological processes, which has been proposed by multiple groups (Li et al., 2015), has so far been limited by the availability of *in vitro* data at the right scales. To date, there has been little effort to model the cell-cell and cell-microenvironment interactions reflected in physiologically relevant, organotypic tissue models. Bioengineered constructs provide for the first time *in vitro* data of sufficient biological complexity to be used in predictive systems biology models of human response; thus, we propose that these constructs are integral to the successful development of such models.

## 2 Defining the vision

### 2.1 Towards the replication of human physiology

The central theme of the workshop was to determine how best to apply the tools of systems biology to organotypic cell cultures in order to achieve a non-reductionist model system that is predictive of human responses. There was the understanding that computational modeling without inputs from empirical data derived from physical models has limited value, yet at the same time it was acknowledged that computational modeling efforts have traditionally been too “phenotypic” in replicating mechanisms of action. Valuable but more focused computational tools have been developed to address the specific needs of toxicology risk assessment and screening therapeutic compounds. Furthermore, a gap between approaches intent on modeling molecular/cellular function on one hand, and tissue/organ function on the other, prevents effective use of the increasing amount of data collected from more biologically complex and relevant systems to enable prediction of the human physiological response to drugs and toxicants.

**Fig. 3:**
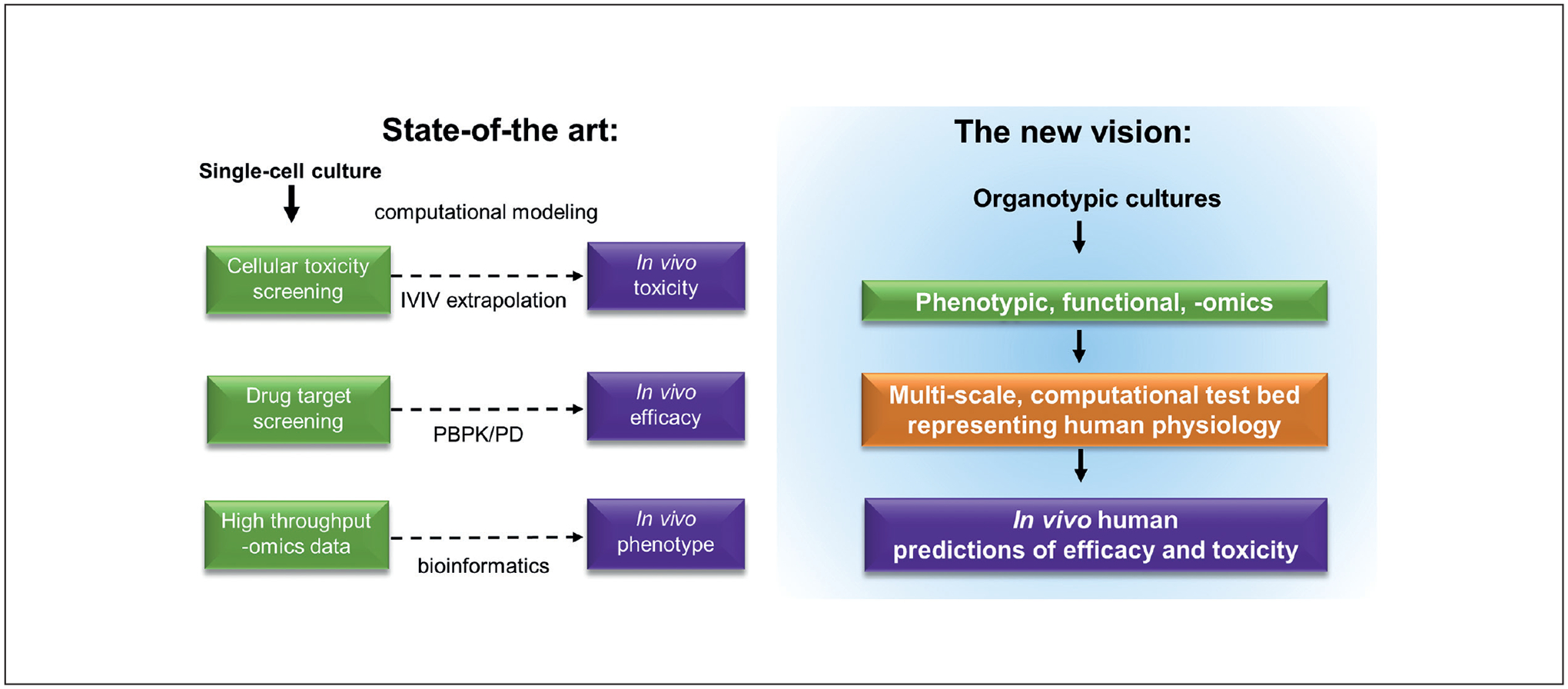
The proposed vision of a computational test bed The proposed vision of a computational test bed leverages tissue-level data from organotypic cultures as well as intracellular data to replicate human physiological response across scales and achieve more accurate predictions.

The proposed solution was a comprehensive computational test bed, capturing the knowledge of all pathways and cellular interactions, and able to accurately predict an organism’s response from key pieces of empirical information describing how a bioactive agent perturbs the system (Fig. 3). This was envisioned as “*a multiscale computational test bed that replicates human physiology*” to capture the breadth of potential applications and minimize the hurdles associated with specific applications for pharmacology or toxicology. Tissue-level measurements from *in vitro* organotypic constructs will serve as the backbone information for the development of the computational test bed. Conversely, with sufficient clinically relevant input and after validation of its predictivity, the computational model needs to inform which data should be obtained from *in vitro* constructs to get “better” data, not just more data. “Better” data are the selected subset of empirical biomarkers that are predictive of health impact upon exposure to a substance. The value proposition for the vision is improved accuracy of IVIVE, reduced cost and time of testing, higher throughput, and ultimately, the ability to handle complex mixtures for both toxicity and polypharmacy applications (Bulusu et al., 2016; Kongsbak et al., 2014).

It is critical, however, that the unperturbed *in vitro* system looks as much like normal healthy tissue as possible, because initial conditions will impact how accurately a system manifests outcomes due to perturbations, particularly long-term outcomes. Thus, while disease modeling is an ultimate goal for this effort, an unmet and immediate need and a key component of this technology is a robust definition of “normal” physiology and the range of parameters that represent “normal”, so that an effect (adverse or therapeutic) can be defined in terms of a perturbation of the system from its normal state.

### 2.2 Benefits of the proposed approach

Successful implementation of the computational test bed would have paradigm-shifting impacts on a number of fields. Toxicologists have attempted to predictively model xenobiotic responses based on chemical structure and a small subset of *in vitro* assays (Mahadevan et al., 2011). It is likely that a computational test bed emulating a physiologically intact organism would be more biologically relevant and evidence-based with opportunities for improved predictivity and decreased dependence on animal studies.

In a clinical context, a reliable comprehensive model including a wealth of molecular pathways and the networks through which they interact will inform novel insights into disease processes. Mechanistic models of pathophysiology will lead to novel therapeutic target identification. Efficient target validation could be carried out *in vitro*, and animal models could also be largely replaced for both safety and efficacy screening in preclinical drug development. The ultimate aspiration would be clinical trials conducted *in silico* with associated saving in time and money. Here, the ability to produce patient-and population-specific organs-on-chips with patient- and healthy subject-derived iPS cells will be an enabling technology, providing the means to define the inter-individual variability in responses to drugs and toxicants and appropriate parameters for *in silico* modeling thereof.

Moreover, precise definition of key pathway perturbations could lead to valuable *in silico* modeling of rare diseases, enabling the identification of potential targets and therapeutics without exhaustive screening, and thus lowering the significant economic barriers associated with developing therapeutics addressing rare diseases. By the same token, sensitive populations such as children could be better protected by more accurate risk assessment. Information derived from the model could also be used to define the most relevant endpoints for epidemiological studies, possibly even identifying biological pathway perturbations that occur long before diseases present in the clinic and therefore addressing the onset of disease. The envisioned *in silico* test bed represents a realistic technology path towards addressing existing and formidable barriers to improving human health by predicting chronic effects or the effects of co-morbidities and polypharmacy (the simultaneous use of multiple drugs). This approach would ultimately shift the focus of health research from fighting disease to promoting wellness (Hood and Price, 2014).

## 3 Barriers to success

### 3.1 Validating organotypic cell cultures and endpoints

The field of engineered cell culture systems is advancing from feasibility demonstration to validation, which is by no means straightforward. The assumption that human-derived cells represent a superior (more predictive) model for human responses than animal cells (and intact animal models) has not been addressed quantitatively in a systematic way across multiple culture platforms. Validation against existing models may initially require the use of species-matched cells (e.g., using murine cells in a model construct and validating against live mouse studies), although some believe that animal models are not appropriate references for validation of human predictivity (Hartung, 2008). Another significant issue is verifying the extent to which organotypic cultures manifest the emergent properties of disease/toxicity. Emergent properties of complex systems are those properties that cannot be ascribed to or predicted from the properties of individual constituents acting in isolation. Often, full manifestation of a pathological phenotype involves multiple interacting physiological systems that are extremely difficult to recapitulate, even in organotypic culture.

The ability to predict clinical outcomes is considered by some the most suitable reference for validation. However, identification of clinically relevant endpoints remains a challenge not only in the translation from a cellular endpoint to a clinical endpoint, but even more fundamentally, in the identification of clinical endpoints associated with unmet clinical needs. Clinical data are limited to begin with, and even clearly defining “disease” is difficult given the aforementioned spectrum of normal, adaptive, and pathological physiology. Current disease classifications are typically limited in their ability to appropriately stratify a disease based on clinical presentation, adequately stage a disease based on how far along a patient has progressed (and where they are headed), or estimate the rate of progression (velocity) of the disease. Efforts towards modeling disease processes, from the clinical domain back towards the underlying physiology and eventual molecular processes, will be critical in the identification of the endpoints needed to qualify *in vitro* systems and the *in vitro* endpoints to be measured for accurate predictive modeling.

An additional aspect that impacts the translatability of *in vitro* systems being used as models are concentrations of the drugs and even the basic maintenance media in the systems. Often these are quite different from corresponding *in vivo* or clinical data, making the baseline control state and perturbations difficult to replicate, thereby creating challenges in making correlations and predictive modeling. From a regulatory standpoint, regulatory conservatism will be a considerable hurdle to overcome, and it will require robust, consistent, and validated data to engender the trust of stakeholders and regulators.

### 3.2 The next frontier in computing?

Biological systems are intrinsically more complex and uncertain than engineered systems. In biological systems, variable scale dynamics and pathobiology only serve to contribute further to their complexity. The computing resources required to simulate and predict accurately the time-dependent behavior of human biology simply do not exist at present. This modeling cannot be carried out even at the individual cell level.

Consider gene regulation within a single cell. The modeling involves huge numbers of variables and stochasticity. The scale of the problem can be seen in the case of compressed models of gene regulation: if one builds a discrete-time stochastic gene regulatory model with 20 genes and assumes binary states (“expressed” or “unexpressed”), then the probability transition matrix is 2^20^×2^20^. In this example, interest is in the steady state distribution of gene expression. This is only for one cell. When multiple cell interactions are taken into account, the complexity increases considerably. At the other extreme, there are continuum-based (partial differential equation, PDE) models of organs, fluid dynamics, chemical transport/reaction/diffusion, and tumor growth (Deisboeck et al., 2011).

**Fig. 4:**
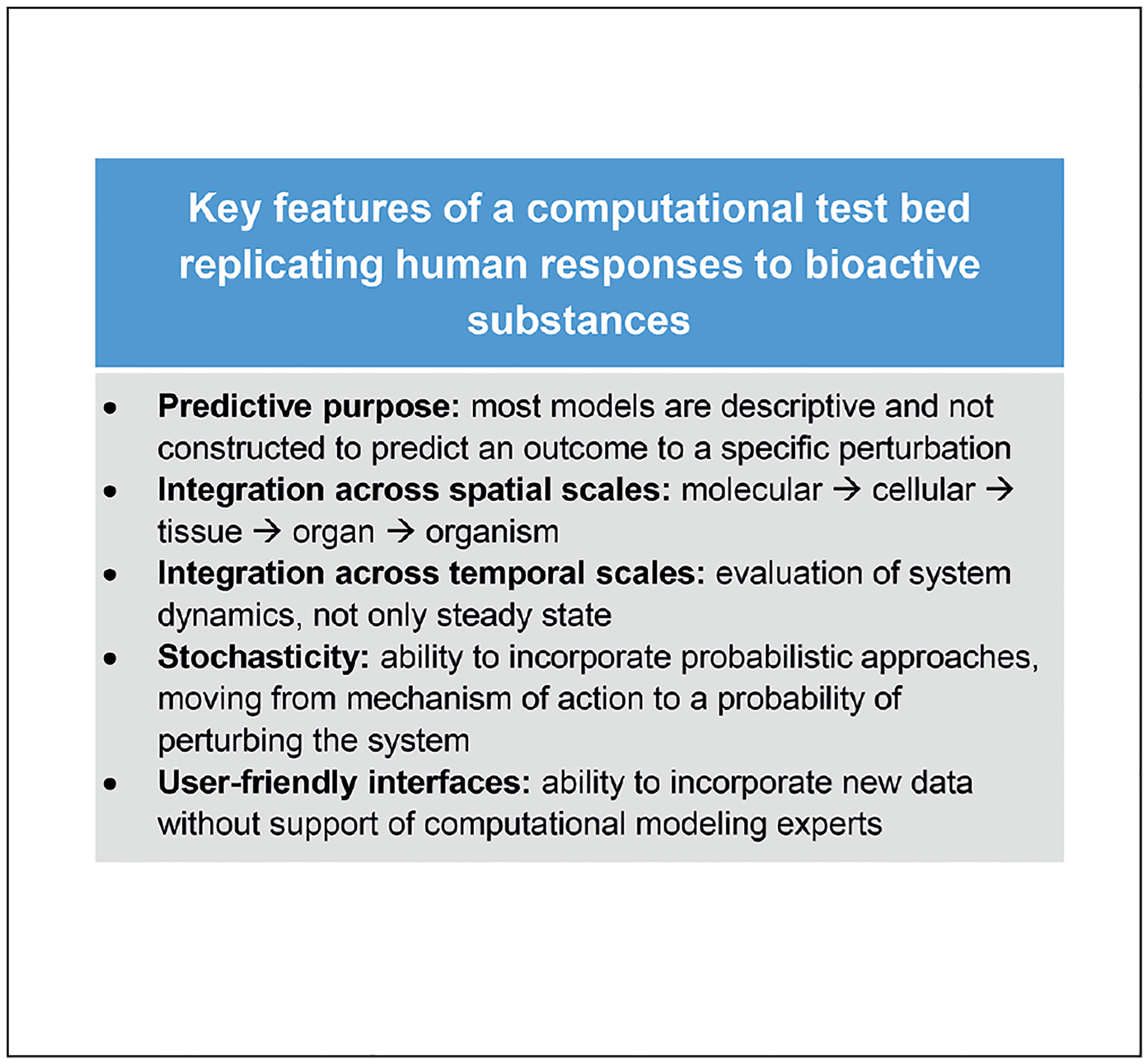
Features to be incorporated in the development of the computational test bed

Bridging the cellular and organ scales is a challenge both mathematically and computationally; determining the uncertainty in predictions across scales adds further complexity, and advances in uncertainty quantification in multiscale methods will need to be made. No computational test bed exists that comprises all the needed features, as listed in Figure 4. Therefore, replicating human biology may well be the next frontier in computing.

### 3.3 Need for collaboration across disciplines

**Fig. 5:**
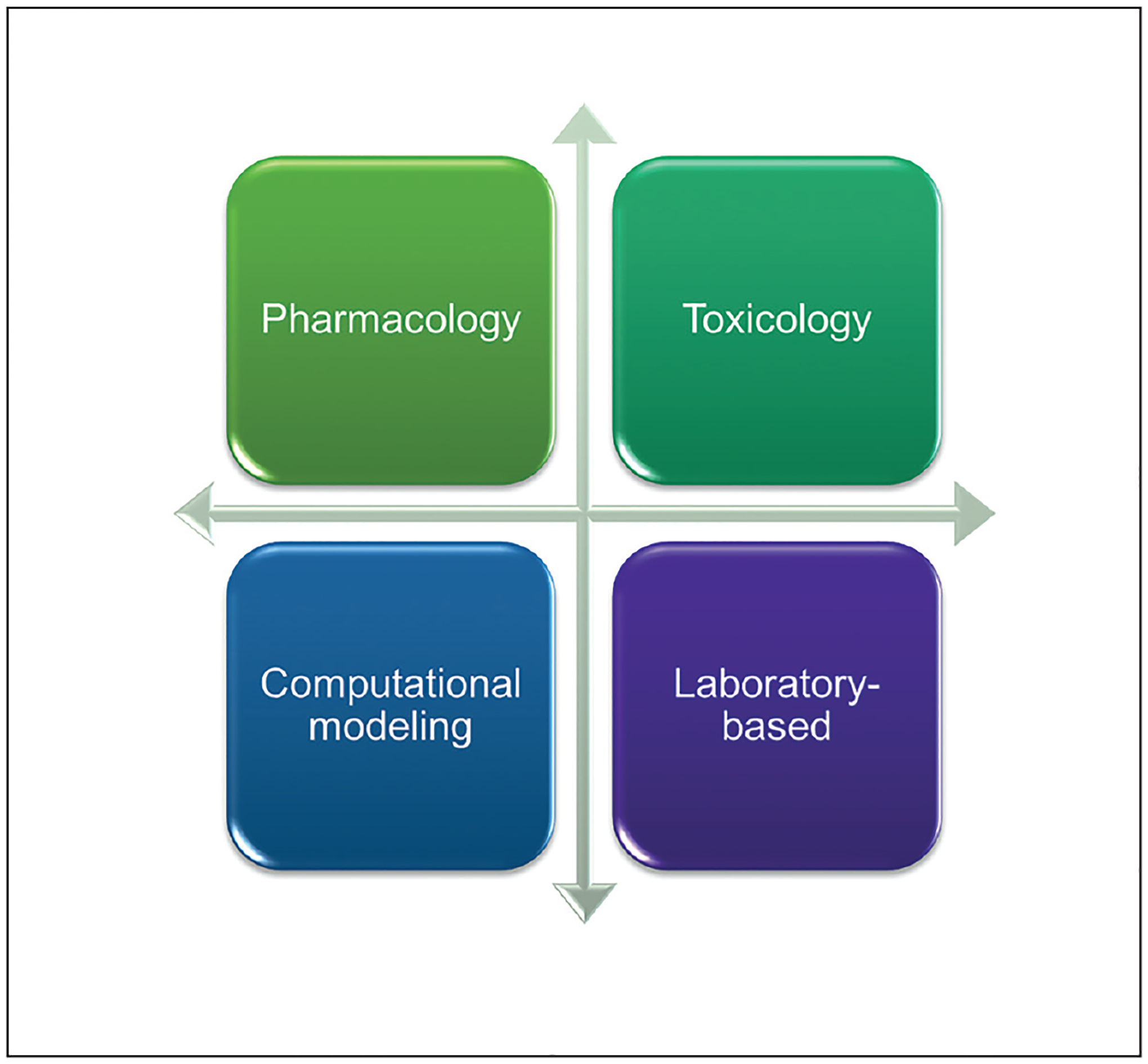
Disciplines and approaches represented in the workshop

Because of the multiple domains of biology involved and the huge computational power required, achieving the vision will require a truly multidisciplinary effort including molecular biologists, geneticists, cellular biologists, physiologists, pathologists, clinicians, information scientists, computer scientists, and engineers. This workshop began addressing this need for cross-disciplinarity by deliberately inviting a diverse group, representing equally the four sectors outlined in Figure 5.

The most salient point raised in the discussion of this topic was the need for sustainable collaboration. Personnel certainly need to possess education across disciplines, but more importantly, group interaction needs to be maintained over time to achieve meaningful progress across disciplinary lines. This will only happen if participants believe that the relevant stakeholders are in it for the long haul and effort is managed and financially supported by long-term commitments.

## 4 Roadmap for the future

Advances in the development of bioengineered 3D multicellular constructs provide *in vitro* data of sufficient quality and depth to be used in predictive systems biology models of human responses. In order to leverage this new source of data to achieve a qualitatively new and improved translation of biological data to human health, the integration of next-generation computational modeling and state of the art cellular screening platforms is key. Both components need to further advance toward this goal.

### 4.1 Experimental approaches

A diversity of *in vitro* tissue engineered models presently exist that capture specific elements of organ functionalities depending on their design. For example, spheroid-type constructs capture heterotypic cell-cell contacts and can (in some cases) benefit from cell-driven self-assembly, whereas nanoporous membrane-based constructs are generally needed to recapitulate tissue interfaces. Integration of multiple organs in one system is a challenge addressed by a specific line of research (Abaci and Shuler, 2015; Oleaga et al., 2016). It is possible, however, to capitalize on the diversity of approaches and maintain the modularity of the engineered constructs, as long as a common computational platform is developed so that the data from these different constructs, as well as data available from clinical studies, can be integrated across scales.

The scientific areas of inquiry to which novel organotypic cell cultures would add the most value would be those in which space (e.g., 3D space within a tissue) and time are central to the hypotheses being tested. Broadly speaking, these would fall into the areas of development, exposure, and disease. In the case of development, heterotypic cell interactions and cell-environment interactions are critical for recapitulating the process of development *in vitro*. The longer-lived cultures enabled by certain platforms would also benefit the study of development (and the perturbation thereof by environmental exposures) as well as enable modeling of “mature” tissues for exposure studies. Longer-lived cultures would also enable studies of cumulative effects of toxicants over time and enable identification of the threshold exposures that can trigger the transition from adaptive response to pathology, both of which are largely neglected by conventional toxicology at present. The same advantages (time, tissue orientation) would increase the sophistication of disease models *in vitro*, particularly for studying metastasis, as well as progressive disease spectrum states, e.g., fatty liver and non-alcoholic steatohepatitis (NASH).

### 4.2 Probabilistic modeling across scales

The central challenge is how to build a computational test bed that provides accurate predictions of *in vivo* human outcomes using data from organotypic culture models. For example, physiologically rich datasets from organotypic models may augment qualitative descriptive pathway models such as stochastic activity networks (Mounts and Liebman, 1997; Tsavachidou and Liebman, 2002) by providing a means to test hypotheses and introduce quantitative information. This requires not only good computational models, but also determining the appropriate experimental readouts to adequately describe processes occurring at molecular, cellular, organ, and whole body scales. Probabilistic models that capture the stochasticity inherent in complex systems are the most predictive for complex cellular behaviors associated with disease and drug targets such as proliferation, migration and inflammation (Stokes et al., 2015). On the other hand, physiologically based pharmacokinetic (PBPK) models based on deterministic approaches are well suited to describe absorption, distribution, metabolism and excretion.

A computational test bed replicating human physiology will likely be a hybrid of deterministic and probabilistic models. Pieces of such an integrated approach exist now as free-standing computational models, but they have yet to be integrated for this purpose. One approach to developing the test bed would be to build upon the existing framework of physiologically based pharmacokinetic/pharmacodynamic models (PBPK-PD) that describe organ- and whole body-level processes via hierarchical integration of probabilistic models.

A chief barrier is the lack of a common language and framework to meaningfully integrate knowledge across scales. Further, the readouts of current PBPK-PD models tend to be single drug targets (i.e., a receptor or enzyme); future models should represent perturbed networks/pathways. In short, the goal is to move PD and toxicodynamics models from single molecular initiating events to dynamic network perturbations. Carefully designed organotypic cultures are enabling the generation of the data required to describe these networks of events.

### 4.3 Computational tools/methods

A systems biology approach to the development of the computational test bed will enable predictive biological insights from complex datasets, as well as application of information control methods, achieving objectives such as classification (diagnosis) and control (therapy) in an optimal manner while relying on a mechanism-based framework (as opposed to purely empirical methods).

As noted previously, modeling biological systems faces huge computational obstacles owing to network complexity, stochasticity, and the need to apply pattern recognition and control algorithms. Model and data reduction methods reduce the dimensionality of the space but must be applied with minimal loss of relevant biological information. This allows simulation with reduced computational power required (Ivanov et al., 2007). There is a trade-off here: reducing dimensionality increases model stochasticity because it puts relevant variables outside the model.

In addition to internal model stochasticity, modeling complex systems faces a second kind of uncertainty, that being the inability to obtain accurate estimates of model parameters. The result is an *uncertainty class* of models, one for each possible vector of values for the parameters. Determination of optimal diagnoses and therapies will have to take this uncertainty into account, the overall task being framed as a constrained optimization problem. Optimal classification (Dalton and Dougherty, 2013), optimal control (Pal et al., 2009), optimal filtering (Dalton and Dougherty, 2014), and optimal experimental design (Dehghannasiri et al., 2015) under model uncertainty will likely play major roles in the path forward.

Methods need to be developed to integrate new types of data into PBPK/PD models. This will involve improved experimental design, computational algorithms, and new types of modeling, for instance, the use of hybrid systems that merge the continuous differential equations of interaction/transport at higher levels with the discrete state transformations typically used in modeling biological pathways.

Some efforts have been made to this end. Computational modeling of physical phenomena can be leveraged to accurately describe exposure. For example, computational fluid dynamics-based description of compound deposition in the respiratory tract (Frederick et al., 2002) yields improved accuracy in the description of absorption for toxico-pharmacokinetic models (Campbell et al., 2014).

The metabolism of a compound within each tissue can be described by enhanced PD (ePD) (Iyengar et al., 2012). In the ePD framework, the drug target network is modeled accounting for enzymatic and metabolic reactions, epigenetic/genetic factors, and/or post-translational modifications. The goal of ePD is to enable prediction of drug/toxicant responses for individual subjects with different genetic and epigenetic backgrounds. In the context of modeling data derived from organotypic systems, ePD could enable the genetic background of the cells used to be taken into account (Thomson et al., 2013).

However, simply combining these elements is not sufficient to produce a “comprehensive” computational test bed. In particular, linking different biological scales in a coherent framework remains a challenge. Even with approaches to achieve efficient computational operations, more powerful computing capacity will be needed so that models can at least be sufficiently large to incorporate basic pathways relating to the etiology of disease, to operate across scales, and to address basic engineering control problems that become extremely computationally intensive in stochastic environments.

There is therefore a need to design appropriate platforms that include specialized architectures: for example, a new operating system may need to be developed that is optimized for computation on biological systems: a “life sciences OS”. Furthermore, because the architecture of the test bed will rely on the mathematics of complex stochastic systems, development of user-friendly visualization tools will be essential to ultimately making the test bed accessible to non-specialists.

### 4.4 Scale of effort required: A Manhattan Project?

Achieving the goal of a multiscale test bed will require considerable public investment on multiple fronts, including technology development, basic and applied research, information infrastructure, and education/training. The eventual overall investment may be on the scale of a Human Genome Project or a small Manhattan Project ($3 billion and $25 billion, respectively). Resources (space, time, people, funds) are needed to develop incubators that foster the biological modeling platforms of the future. Alignment of vision and goals across disciplinary sectors is a key hurdle for advancing novel capabilities to real impact.

From a project management standpoint, development of critical milestones and plans to achieve intermediate success and prevent overall failure were suggested. Also, a project toward this goal needs to define testable hypotheses and harmonized evaluation standards.

This significant computational effort would benefit from extreme scale computing, called exascale computing, which would perform with 1,000x improvement over today’s Petaflop computers with a similar size and power footprint. Exascale computing is currently being pursued by the DOE (United States Department of Energy) for energy, nuclear, and national security applications (Russell, 2015); however, advanced computing for predictive biology to improve human health, explored by initiatives such as BAASiC (http://baasic.llnl.gov/), could prove to be the application most beneficial to the public good.

While government funding is needed to drive this approach, other mechanisms exist to advance the field by encouraging sharing of critical information. For example, the pharmaceutical industry could move to treat drug safety assessment as “pre-competitive”, as has occurred in the food industry (Stubbs, 2014; Whitworth, 2015). It would be highly valuable to the advancement of predictive modeling of human response if the wealth of data collected from safety assessments of therapeutics were made publicly available, since this includes data spanning *in vitro* assays to human clinical data for a wealth of compounds, particularly those that failed to go to market. Industry could elect to make these data public while maintaining compound efficacy data and assays proprietary and therefore maintain their competitive advantage.

In summary, this overview highlights the opportunities, tremendous benefits, formidable barriers, and key steps toward the goal of a computational systems biology approach to translate *in vitro* data to prediction of human response. An initiative supporting the development of this vision will require a considerable public investment; gaining support for it requires raising awareness of its benefit with the general public. Linking the goals of this initiative to containing rising healthcare costs is a promising path towards public/political support for a Manhattan Project-scale investment.
